# Association between per capita sugar consumption and diabetes prevalence mediated by the body mass index: results of a global mediation analysis

**DOI:** 10.1007/s00394-020-02401-2

**Published:** 2020-10-09

**Authors:** Alexander Lang, Oliver Kuss, Tim Filla, Sabrina Schlesinger

**Affiliations:** 1 Institute for Biometrics and Epidemiology, German Diabetes Center, Leibniz Institute for Diabetes Research at Heinrich Heine University Düsseldorf, Auf’m Hennekamp 65, 40225 Düsseldorf, Germany; 2 Institute for Biometrics and Bioinformatics, University Hospital Düsseldorf, Research at Heinrich Heine University Düsseldorf, Moorenstraße 5, 40225 Düsseldorf, Germany; 3 German Center for Diabetes Research (DZD e.V.), München-Neuherberg, Germany

**Keywords:** Diabetes, Ecological study, Mediation, Obesity, Sugar

## Abstract

**Purpose:**

The aim of this study was to examine the mediation of body mass index (BMI) on the association between per capita sugar consumption and diabetes prevalence using country-related data.

**Research design and methods:**

In this ecological study, based on 192 countries, data on per capita sugar consumption were obtained from the Food and Agriculture Organization of the United Nations (FAO), on BMI from the World Health Organization and on diabetes prevalence from the International Diabetes Federation. Data on demography and economic factors were obtained from the Central Intelligence Agency, the United Nations and the FAO. Multiple linear regression analysis was performed to investigate the association between per capita sugar consumption and diabetes prevalence, and mediation analysis to detect the mediated percentage of BMI on this association.

**Results:**

Each increase of 100 kcal/day per capita sugar consumption was associated with a 1.62% higher diabetes prevalence [adjusted β-estimator (95% CI): 1.62 (0.71, 2.53)]. Mediation analysis using BMI as the mediator demonstrated an adjusted direct association of 0.55 (95% CI: − 0.22, 1.32) and an adjusted indirect association of 1.07 (95% CI: 0.54, 1.68). Accordingly, the BMI explained 66% (95% CI: 34%, 100%) of the association between per capita sugar consumption on diabetes prevalence.

**Conclusions:**

These findings indicate that the association between dietary sugar intake and the occurrence of diabetes is mediated by BMI to a large proportion. However, it seems that other mechanisms may explain the association between sugar consumption and development of type 2 diabetes.

**Electronic supplementary material:**

The online version of this article (10.1007/s00394-020-02401-2) contains supplementary material, which is available to authorized users.

## Introduction

There is an ongoing debate whether dietary sugar intake has an impact on the development of type 2 diabetes (T2D). In the last decades, per capita sugar consumption increased, particularly in developing and emerging countries [1], due to demographic growth, rising income and increasing availability of sugar containing products [2]. On the contrary, per capita sugar consumption was stable or decreasing in developed countries [1, 3]. Nevertheless, per capita sugar consumption still remains high in western civilizations [1, 4]. Along with the high intake of dietary sugar, there has been a large increase in the incidence of T2D, which is a heterogeneous disease and caused by environmental as wells as genetic components. Several risk factors are known, including, e.g., age, overweight or obesity, or family history of diabetes [5, 6]. During the last decades, lifestyle factors, including dietary behaviour have been recognised as important predictive factors for the development and progression of T2D [7]. For dietary factors, high certainty of evidence was observed for lower incidence of T2D for a healthy dietary pattern, including high intake of fibre from whole grain products and low intake of red meat and sugar sweetened beverages [8]. In this context, a high intake of dietary sugar may contribute to an overload of calorie intake and may lead to obesity [9], which is a causal risk factor for T2D [10]. Furthermore, direct pathophysiologic mechanisms due to glycaemic effects independent of overweight and obesity via, e.g., an increase of liver fat content or specific adipose tissues [11, 12], might explain the association between dietary sugar intake, particularly fructose and sugar sweetened beverages intake, and risk of T2D [13–16].

However, findings from observational studies on the relation between total dietary sugar intake and T2D incidence were inconsistent. While an increase of sugar sweetened beverage consumption was consistently associated with T2D [8, 17, 18], the evidence regarding the association between the intake of mono- and disaccharides regarding development of T2D is insufficient [8]. In a systematic review and meta-analysis, associations between total dietary sugar intake and T2D were summarized and higher intake of total sugar was inversely, but imprecisely estimated, associated with incidence of T2D [19]. There was heterogeneity between the studies, and risk estimates pointed to an inverse direction [20] or null associations [21–25], respectively. In addition, the meta-analysis summarized findings between fructose and sucrose intake and risk of T2D and reported inverse relationships [19], but again, the results of the single studies were heterogeneous and pointed to different directions. Moreover, some of the studies indicated that the body mass index (BMI) might bias this relationship [21, 24, 25]. Nevertheless, findings of observational studies need to be interpreted with caution, since dietary sugar intake is measured in general by self-reports of the participants, which is prone to bias, because participants, particularly overweight participants, tend to underreport their true intake of unhealthy food [26–28]. Findings from ecological studies, based on aggregated estimations of per capita sugar consumption showed a positive correlation with diabetes prevalence at country level [29–31]. However, in these studies, the impact of BMI on the association between dietary sugar intake and diabetes prevalence was not considered yet. Thus, we conducted a global ecological study to investigate the direct association between dietary sugar consumption and T2D and the indirect association mediated via the BMI using aggregated data from single countries.

## Methods

### Study design

**Fig. 1:**
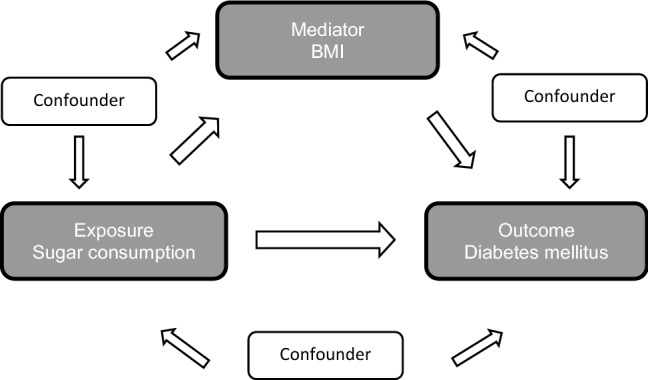
Chart of the causal pathways regarding the association between sugar consumption and diabetes prevalence

This is a mediation analysis based on an ecological study design, including geographical group-level country data. The study is reported according to the Guidelines for Accurate and Transparent Health Estimates Reporting (GATHER) [32], and Strengthening the Reporting of Observational Studies in Epidemiology—Nutritional Epidemiology (STROBE-nut) [33]. Data from several official data-collection sources were obtained on: per capita sugar consumption (exposure), diabetes prevalence (outcome), and BMI (mediator). Since this is a mediation analyses, we considered the “causal pathways” between dietary sugar intake and obesity [9], and consequently, between obesity and incidence of T2D [10], and thus decided to assess our data in an a priori defined time-sequence of 5 year intervals. In addition, we selected available potential confounders a prior [34]: mean age [35], per capita gross domestic product (GDP) as a socioeconomic variable [36], per capita fat intake [8], total energy supply [37] and percentage of rural population [38]. All considered data were based on estimations. For sensitivity analysis data on prevalence of overweight (BMI ≥ 25 kg/m^2^ − < 30 kg/m^2^) and obesity (BMI ≥ 30 kg/m^2^) were collected. All countries with available data on at least half of the considered variables were included in this study. Finally, for the present analysis data from 192 countries were selected (Supplement: Fig. 1).

### Data collection

Data on per capita sugar consumption adjusted for exports and any kind of non-human consumption were obtained for the year 2007 from the Food and Agriculture Organization of the United Nations (FAO) that received the estimated data mainly from national statistical offices [39]. Per capita sugar consumption contains the food supply of raw centrifugal sugar of cane as well as beet, refined sugar, sugar confectionary and sugar flavoured products per person in kilocalories per day [39]. Mean BMI and the prevalence rates of overweight and obesity were collected for the year 2012 from the WHO [40]. Data on age-adjusted diabetes prevalence were obtained for the year 2017 from the International Diabetes Federation (IDF) which extracted the data from peer-reviewed articles, national health surveys and other official sources [41]. Matched to the acquisition of the exposure, data on all potential confounders were also obtained from 2007. Therefore, country-level per capita GDP was received from the statistics division of the United Nations (UN) [42]. Further, data on mean age were obtained from the Central Intelligence Agency (CIA) [43], and data on rural population, per capita fat intake and total energy supply per day were collected from the FAO [39, 44]. Energy supply is defined as the average per capita caloric availability, which means that this number does not necessarily indicate the real amount of calories that was actually consumed [39]. Countries were categorized as continents according to the classification of the UN [45]. For a sensitivity analysis, we collected the most recent data on per capita sugar consumption [39] and potential confounders (2013) [39, 42–44], BMI (2016) [40], and diabetes prevalence (2017) [41] from the same organizations.

### Statistical analysis

Pearson’s correlation coefficients were calculated to describe the correlations between per capita sugar consumption and diabetes prevalence, as well as between per capita sugar consumption and BMI, and between BMI and diabetes prevalence. Multiple linear regression analysis, adjusted for age, per capita GDP, per capita fat intake and total energy supply and rate of rural population, was accomplished to estimate the association between per capita sugar consumption and diabetes prevalence. We performed the mediation analysis by applying the method suggested by VanderWeele [46]. For this, the following criteria must be fulfilled: first, the exposure (per capita sugar consumption) has to be associated with the outcome (diabetes prevalence). Second, the exposure (per capita sugar consumption) has to be associated with the mediator (BMI), and third, the mediator (BMI) has to be associated with the outcome (diabetes prevalence). If the criteria are fulfilled, mediation analysis can be conducted to estimate the direct association of per capita sugar consumption [per 100 kcal/day] on diabetes prevalence and the indirect association of this relation via the “causal pathway” of obesity (Fig. 1). If a total association between exposure and outcome exists, and the direct association is low, it can be interpreted that the association is mediated to a large proportion by the mediator. In contrast, if the indirect association is low, it implicates that the potential mediator has little or no mediating impact on the association between exposure and outcome. Moreover, the mediated percentage can be calculated, which is defined as the quotient of the indirect association in relation to the total association, to estimate the percentage impact of BMI on the association between per capita sugar consumption and diabetes prevalence [46].

Standard multiple imputation methods were used to account for missing values, where for per capita sugar consumption *n* = 22 were missing, for diabetes prevalence *n* = 4, BMI *n* = 2, age *n* = 1, fat intake *n* = 24, total energy *n* = 22 and rural population *n* = 16. All available variables were used for imputation and 1000 data sets were generated. The mediation model was calculated for each imputed data set, and we reported the median value from the 1000 model fits as the estimated parameter together with the 2.5% and 97.5% percentiles as the respective non-parametric 95% confidence interval (CI). If the 95% CI does not include the null-value, the findings are estimated precisely and can be interpreted as “statistically significant”. We chose this simultaneous application of multiple imputation and a non-parametric bootstrap procedure as there is still methodological debate on how confidence intervals for mediation parameters should be calculated. In addition, by combining the two approaches it is guaranteed that the complete statistical variability in the data is properly accounted for, yielding conservative confidence intervals that are rather too long than too short. Statistical analyses were performed in R 3.6.1 and SAS, Version 9.4 by the MI and the CAUSALMED procedure.

## Results

After performing multiple imputation, data on 192 countries were assessed for analyses. The characteristics for the included countries are shown in Supplement: Tables 1–7 allocated by regions defined by the UN [47]. Mean (SD) per capita sugar consumption was 191 ± 122 kcal/d. Highest per capita sugar consumption was seen in Central America (428 ± 55 kcal/d), Oceania (407 ± 86 kcal/d) and Eastern Europe (386 ± 80 kcal/d). Mean diabetes prevalence was 8.5 ± 2.8%, whereas highest diabetes prevalence was observed in Northern Africa (12.8 ± 5.7%), Central America (12.6 ± 2.0%) and Western Asia (11.0 ± 4.0%). Global mean BMI was 24.0 ± 2.3 kg/m^2^, with the highest mean BMI in Northern America (28.4 ± 1.0 kg/m^2^), Central America (27.4 ± 0.6 kg/m^2^) and Western Asia (27.3 ± 1.5 kg/m^2^).

Findings showed a positive correlation between per capita sugar consumption and diabetes prevalence (Pearson: *r* = 0.37 (95% CI: 0.24, 0.49), *p* < 0.01). After stratification by region, this positive trend was observed for Asia (*r* = 0.56 (95% CI: 0.17, 0.80), *p* = 0.01) and Africa (*r* = 0.42 (95% CI: − 0.03, 0.73), *p* = 0.06), but not for Oceania (*r* = − 0.34 (95% CI: − 0.97, 0.86), *p* = 0.67) and Europe (*r* = − 0.26 (95% CI: − 0.64, 0.23), *p* = 0.30) (Supplement: Table 10). Similar findings between per capita sugar consumption and diabetes prevalence were observed after stratification by income classification, showing positive correlations for lower middle and upper middle income countries, and null associations for low and high income countries regarding the association between per capita sugar consumption and diabetes prevalence (Supplement: Table 11).

**Fig. 2:**
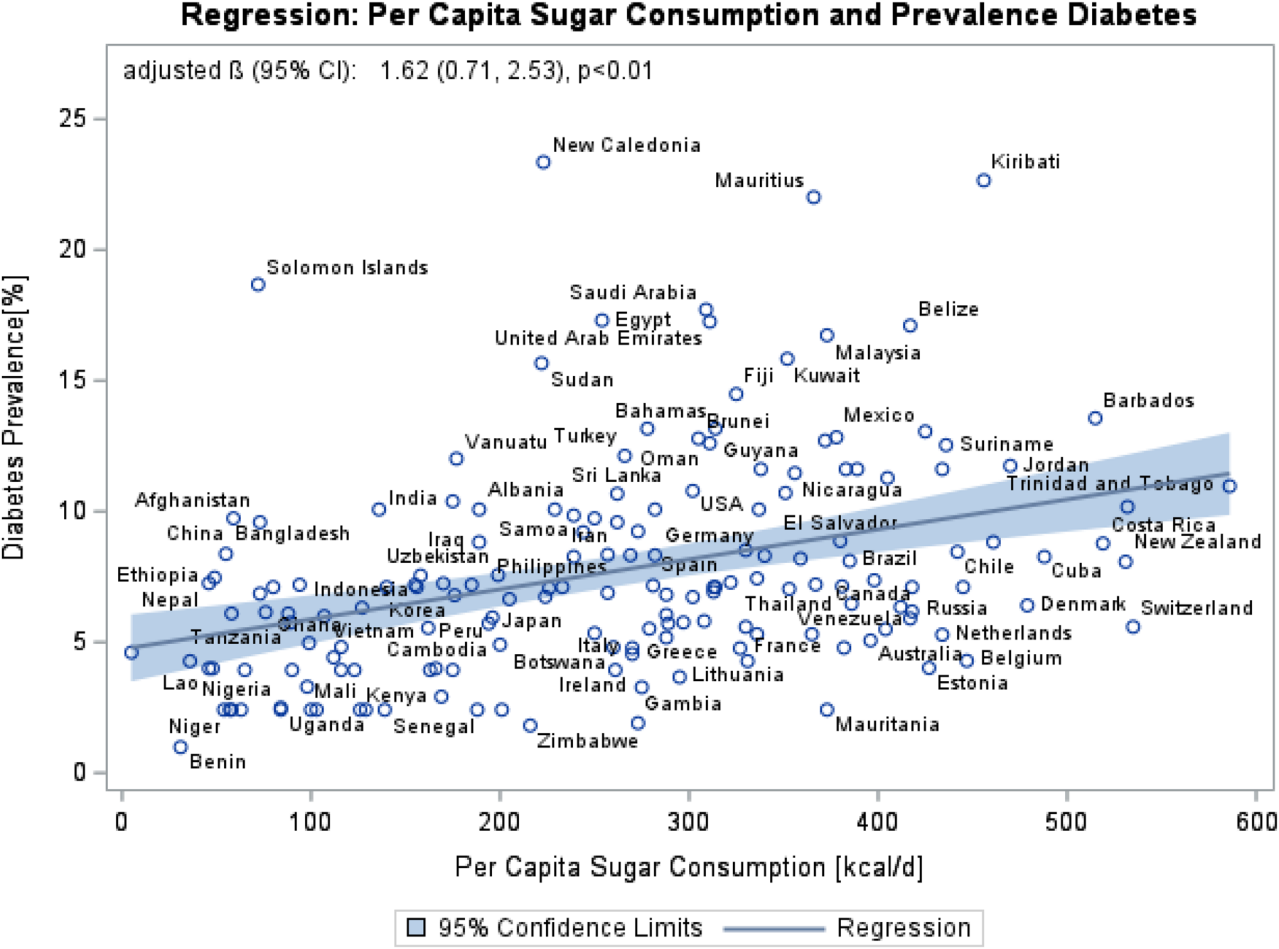
Linear regression of per capita sugar consumption (2007—FAO) and diabetes prevalence (2017—IDF)—including 192 countries

There was also positive correlations between per capita sugar consumption and mean BMI (*r* = 0.67 (95% CI: 0.58, 0.75), *p* < 0.01) and mean BMI and diabetes prevalence (*r* = 0.58 (95% CI: 0.46, 0.68), *p* < 0.01), respectively (Supplement: Figs. 1 and 2). Correlation coefficients between exposure or outcome with potential confounding variables were positive for mean age, per capita GDP, per capita fat intake and total energy or inverse correlations for rural population (Supplement: Table 12).

Results of univariate linear regression models indicated a positive association of per capita sugar consumption with diabetes prevalence (β (95% CI): 1.42 (0.72, 2.13), *p* < 0.01) and BMI (β (95% CI): 1.18 (0.91, 1.45), *p* < 0.01) (Supplement: Fig. 2), respectively. BMI was positively associated with diabetes prevalence (β (95% CI): 1.16 (0.74, 1.51), *p* < 0.01) (Supplement: Fig. 3). Adjustment for BMI weakened the association between per capita sugar consumption and diabetes prevalence (β (95% CI): 0.12 (− 0.83, 1.07), *p* = 0.81), indicating that BMI is a mediator. In the multiple linear regression model adjusted for age, per capita GDP, per capita fat intake, total energy and rural population, each increase of per capita sugar consumption per 100 kcal/day was associated with 1.62% higher diabetes prevalence (β (95% CI): 1.62 (0.71, 2.53), *p* < 0.01) (Fig. 2). The findings of our mediation analysis showed an adjusted direct association of 0.55 (95% CI: − 0.22, 1.32) and an adjusted indirect association of 1.07 (95% CI: 0.54, 1.68) regarding the relation between per capita sugar consumption and diabetes prevalence. According to this, the mediated percentage of BMI on this association was 66% (95% CI: 34%, 100%) after adjusting for covariates (Table 1). In reverse, about one third of the total association between sugar consumption and prevalence of diabetes could be explained by other mechanisms.

**Table 1:** Mediation analysis for BMI on the association between per capita sugar consumption per 100 kcal/day and diabetes prevalence

	Model 1 (crude)	Model 2 (adjusted)*
	β [95% CI]	β [95% CI]
β_total_	1.42 [0.72, 2.13]	1.62 [0.71, 2.53]
β_direct_	0.13 [− 0.67, 0.87]	0.55 [− 0.22, 1.32]
β_indirect_	1.28 [0.71, 2.00]	1.07 [0.54, 1.68]
% mediated	90.7% [48.6%, 100.0%**]	66.1% [34.4%, 100.0%**]

*Adjusted for age [years] (2007–CIA), per capita gross domestic product [US$] (2007–UN), total energy [kcal/day] (2007–FAO), per capita fat intake [g/d] (2007–FAO) and rural population [%] (2007–FAO)

**Bootstrap 95% CI cut off by 100%

Sensitivity analysis showed similar results when using the most recent data that were available (Supplement: Fig. 4 and Table 8). Performing mediation analysis using the prevalence of overweight or obesity as mediator, the adjusted mediated percentage was 40% (95% CI: 15%, 78%) for overweight, and 51% (95% CI: 23%, 87%) for obesity, respectively (Supplement Table 9).

## Discussion

The findings of our ecological study showed an association between dietary sugar intake and prevalence of diabetes and suggested that the total association can be explained by about two thirds via BMI. In consequence, there might be other mechanisms that explain the association between dietary sugar intake and diabetes.

Regarding the impact of dietary sugar intake on the development of T2D, the evidence is inconclusive and findings are controversial [8, 19]. Compared to other ecological analyses that have examined the association between per capita sugar consumption and diabetes prevalence, we received similar results regarding the relationship between dietary sugar intake and diabetes prevalence [29–31]. Moreover, several studies indicated that consumption of sugar sweetened beverages was associated with weight gain and increased T2D risk [8, 17, 18]. However, findings of observational studies reported an inverse relation [20] or no associations [21–25] concerning the association between total dietary sugar intake and risk of T2D. Nevertheless, some of these studies suggested an inverse association which disappeared after adjusting for BMI [21, 24, 25].

These observations indicate that reporting bias dependent on BMI status cannot be ruled out. Since nutrition data is mainly based on self-reports of participants, it is likely that intake of unhealthy food, including sugar containing products, can be biased due to measurement errors, because especially, overweight participants tend to underreport their true intake [26–28]. As a result of this, other reliable methods to assess dietary sugar intake, like measuring a sucrose biomarker, e.g., in urine samples, should be used in the future to eliminate recall bias [48, 49]. Some studies already investigated objective sucrose biomarker and found deviations in comparison to dietary sugar intake assessed with Food Frequency Questionnaires (FFQ) or 24-h dietary recalls (24HRs) [49, 50]. In addition, various cross-sectional studies investigated stable isotope ratios of carbon (δ^13^C) and nitrogen (δ^15^N) as potential sugar biomarker in adults measured in hair, capillary finger stick blood and blood serum [51–54]. Findings of these studies indicated that δ^13^C might potentially be an objective biomarker to assess dietary sugar intake, since δ13C values were predictive for the intake of added sugar [52–54], sugar sweetened beverages [51–53], as well as total sugar intake [54]. However, findings concerning δ^15^N biomarker were inconclusive predicting sugar intake. While results in an Alaska native study population showed a strong association between δ^15^N and dietary sugar intake [54], findings of another population did not show any association [53].

The present body of research implies that an increased body weight might be the major explanation for the observed association between dietary sugar intake and diabetes prevalence. A high dietary sugar intake may lead to overweight and obesity which is associated with increased risk of T2D. Since the amount of adipose tissue is enlarged, overweight and obesity increase the secretion of nonesterified fatty acids and different hormones and cytokines, such as leptin, adiponectin and pro-inflammatory cytokines, which are associated with insulin resistance [55, 56]. In addition, obesity can cause a decrease in β-cell function [55], which leads to the development of T2D over time [57]. Moreover, as our results indicated, other possible pathophysiologic mechanisms, independent of the influence of overweight and obesity, might explain the association between dietary sugar intake and the development of diabetes. For example, intake of large amounts of added sugars can contribute to a diet with high glycaemic load which can induce higher glycaemic and insulinemic response [15]. Findings from observational studies have shown that diets with high glycaemic loads were associated with increased risk of T2D [58, 59]. A possible explanation for this observation could be a decrease in insulin sensitivity [60, 61], which can progress to T2D [59, 62]. In addition, different types of sugars might have different effects on insulin resistance and blood glucose control. Recently, a systematic review and network meta-analysis showed that the dietary isocaloric exchange of fructose with glucose had a beneficial effect on insulin resistance [63]. A further explanation for an association between sugar intake and T2D could be the development of non-alcoholic fatty liver disease (NAFLD), which has shown a bidirectional association with T2D [64, 65]. Dietary sugar intake, especially fructose intake, has been linked to an increase in liver fat content and NAFLD [11, 12], which is strongly associated with insulin resistance [66].

Our study had several strengths. This was the first study performing a causal mediation analysis that investigated the impact of BMI as mediator for the association between per capita sugar consumption and diabetes prevalence. Besides this, the used data, including potential economic and health-related confounders, were current and obtained from reliable global organisations. Furthermore, we considered lead time intervals between exposure, mediator and outcome to simulate a prospective sequence between these factors. However, our study had some limitations as well. First, conclusions about causality are hampered due to the ecological study design. Future prospective studies are needed to ascertain more reliable findings regarding the direct association between dietary sugar intake and incidence of T2D based on individual data while using objective biomarkers to assess the true dietary sugar intake. Second, data regarding diabetes prevalence obtained from the IDF also contained individuals with type 1 diabetes or other types of diabetes, which might be approximately around 7–12% and 1–3% in high income countries, respectively [41]. This may affect the results due to, e.g., different body composition compared to individuals with T2D. Overall, the collected population-related data are mainly not based on individual assessments, but on estimations and projections, especially for developing countries due to insufficient opportunity of central data collection. Accordingly, data quality might differ due to differences in surveillance infrastructure between countries. Moreover, supply data as it was used for per capita sugar consumption, fat and total energy intake might not reflect the actual intake of an individual, but only the amount reaching the consumer [39]. Hence, obtained data may differ from the true values [39, 41]. Beyond that, confounding cannot be ruled out, because relevant data, e.g., further dietary factors, physical activity, or smoking, were not available and thus, could not be considered in this analysis. Consequently, additional potential confounders should be considered in future research.

## Conclusions

In this ecological study, per capita sugar consumption was positively associated with diabetes prevalence at a global level. This association was mediated by 66% by BMI, indicating, that a large amount of this association can be explained by BMI, but that also other mechanisms exist that could explain the association between dietary sugar intake and the development of T2D. Causality cannot be proved with an ecological study design, and thus further well-conducted studies are recommended that use individual participant data and valid measurements for their sugar consumption.

## Electronic supplementary material

Below is the link to the electronic supplementary material.

Supplementary file1 (PDF 951 kb)

## Data Availability

Data were extracted from several official data-collection sources, all of which are publicly available and accessible.

## Code availability

Will be provided if requested.
