# Supplementary material for: Association between per capita sugar consumption and diabetes prevalence mediated by the body mass index: results of a global mediation analysis
Source: Eur J Nutr. 2020 Oct 9;60(4):2121–9. doi: 10.1007/s00394-020-02401-2 (PMC8137616; doi:10.1007/s00394-020-02401-2)
Supplement: Supplementary file 1 — Supplementary file1 (PDF 951 kb) [file 394_2020_2401_MOESM1_ESM.pdf]

Online supplemental material

Association between per capita sugar consumption and diabetes prevalence mediated by the body mass index: results of a global mediation analysis

Alexander Lang[1], Oliver Kuss[1,3], Tim Filla[2], Sabrina Schlesinger[1,3]

[1] Institute for Biometrics and Epidemiology, German Diabetes Center, Leibniz Institute for Diabetes Research at Heinrich Heine University Düsseldorf, Auf'm Hennekamp 65, 40225 Düsseldorf, Germany

[2] Institute for Biometrics and Bioinformatics, University Hospital Düsseldorf, Research at Heinrich Heine University Düsseldorf, Moorenstraße 5, 40225 Düsseldorf, Germany

[3] German Center for Diabetes Research (DZD e.V.), München-Neuherberg, Germany

Author of correspondence: Sabrina Schlesinger, Sabrina.Schlesinger@ddz.de

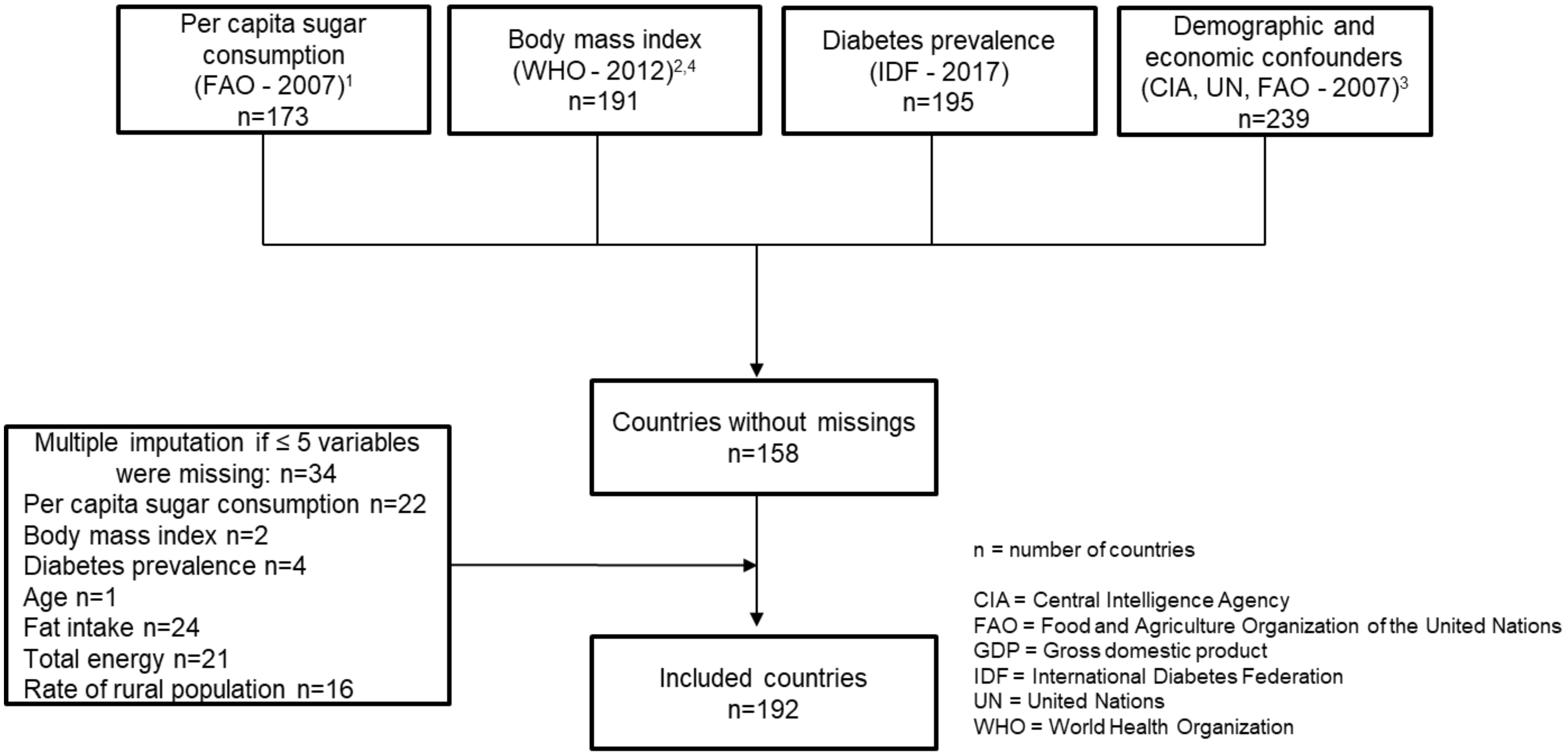

[1] for sensitivity analysis with most recent data: data from FAO - 2013, n=173
[2] for sensitivity analysis with most recent data: data from WHO - 2016, n=191
[3] for sensitivity analysis with most recent data: data from CIA, UN, FAO - 2013, n=239
[4] for sensitivity analysis mediated by overweight and obesity prevalence: data from WHO - 2012, n=191

Supplement: Figure 1: Flow chart of data assessment and preparation

Supplement: Table 1: Characteristic variables by country for Africa, including 53 countries

Country	Per capita sugar intake [kcal/d]	Diabetes prevalence [%]	Mean BMI [kg/m²]	Overweight prevalence [%]	Obesity prevalence [%]	Mean age [years]	Per capita GDP [US$]	Per capita fat intake [g/d]	Total energy [kcal/d]	Rural population [%]	Total population
Algeria	302	6.73	25.2	59.0	24.7	25.5	3935	67.1	3104	35.4	34,166,976
Angola	116	3.94	23.0	24.8	6.8	17.9	3108	47.0	1949	44.2	20,905,360
Benin	31	0.99	23.2	27.2	8.2	17.7	706	46.8	2512	59.2	8,454,790
Botswana	260	4.81	24.2	41.4	17.5	20.9	5714	55.7	2235	41.1	1,875,458
Burkina Faso	54	2.42	21.9	20.7	4.5	16.5	475	54.5	2669	80.9	14,252,029
Burundi	data imputed	6.05	21.4	20.1	4.4	16.7	171	data imputed	1680	89.9	7,862,226
Cabo Verde	201	2.42	24.3	32.3	10.3	20.2	3112	71.8	2549	41.1	475,067
Cameroon	94	7.20	24.1	31.0	9.8	18.9	1216	51.2	2259	44.0	18,730,283
Central African Republic	58	6.10	22.6	24.0	6.4	18.5	410	71.1	1956	61.7	4,198,004
Chad	88	6.10	21.7	21.1	5.1	16.3	779	50.0	2040	73.8	10,818,031
Comoros	data imputed	11.88	24.0	25.1	6.7	18.7	1238	data imputed	1857	72.0	641,624
Congo	185	7.20	22.9	28.6	8.3	16.7	2206	49.3	2513	39.1	3,876,123
Côte d'Ivoire	100	2.42	23.6	28.9	8.7	19.3	1066	54.7	2515	51.8	19,171,250
Dem. Rep. Congo	data imputed	6.10	21.7	23.0	5.6	16.1	287	data imputed	1585	66.7	58,453,687
Djibouti	288	6.05	23.9	36.8	12.3	18.2	996	68.1	data imputed	data imputed	805,456
Egypt	254	17.31	29.1	60.8	29.3	24.2	1662	61.5	3163	57.4	78,232,124
Equatorial Guinea	data imputed	7.78	23.7	24.6	6.8	18.8	15762	data imputed	data imputed	60.9	821,686
Eritrea	data imputed	6.05	20.6	20.0	4.1	17.9	317	data imputed	1587	79.8	2,996,540
Ethiopia	49	7.47	20.4	18.9	3.6	18.0	234	22.6	1952	83.4	80,674,343
Gabon	155	7.20	24.9	37.9	13.5	18.6	8353	58.0	2730	15.3	1,472,565
Gambia	273	1.91	23.6	29.1	8.7	17.8	785	71.8	2345	44.3	1,639,846
Ghana	99	4.97	23.7	29.6	9.4	20.2	1444	43.6	2849	50.7	22,963,946
Guinea	126	2.42	22.7	24.4	6.4	17.7	622	57.8	2529	66.1	9,518,159
Guinea-Bissau	103	2.42	23.2	27.2	7.9	19.1	481	60.4	2288	70.3	1,411,545
Kenya	169	2.92	23.0	23.2	5.9	18.6	839	48.6	2060	78.7	38,705,934

Lesotho	163	3.94	24.8	36.3	14.9	21.1	919	33.7	2468	75.3	1,986,926
Liberia	58	2.42	23.9	28.6	8.6	18.1	270	56.3	2163	40.5	3,461,911
Libya	data imputed	10.43	27.7	64.5	30.0	23.3	10497	data imputed	3144	22.7	5,974,786
Madagascar	65	3.94	21.1	21.5	4.3	17.8	446	27.9	2133	70.9	19,433,520
Malawi	123	3.94	22.6	21.5	4.8	16.7	320	34.6	2127	81.7	13,341,808
Mali	129	2.42	22.7	25.4	7.2	15.9	596	data imputed	2579	68.4	13,651,455
Mauritania	373	2.42	24.7	31.8	11.0	17.1	1013	70.7	2823	59.3	3,202,512
Mauritius	366	22.02	25.1	30.6	9.6	31.2	6605	88.0	2936	57.7	1,233,890
Morocco	381	7.14	25.6	57.5	23.4	24.3	2531	64.8	3230	44.4	31,163,670
Mozambique	98	3.30	22.5	24.4	6.1	17.4	422	36.7	2071	63.9	21,673,319
Namibia	175	3.94	23.9	37.4	15.1	20.2	4203	53.9	2349	63.8	2,006,516
Niger	57	2.42	21.6	20.0	4.5	16.5	292	52.9	2306	83.6	14,685,404
Nigeria	84	2.42	23.1	26.2	7.4	18.7	1828	65.9	2708	52.4	146,339,971
Rwanda	36	4.28	21.8	22.6	4.7	18.6	405	24.2	2054	82.0	9,273,759
São Tomé and Principe	188	2.42	24.6	32.7	10.7	16.2	894	data imputed	2662	39.9	166,297
Senegal	139	2.42	22.8	26.2	7.6	18.7	1230	61.6	2318	58.0	11,687,078
Seychelles	data imputed	10.55	26.4	34.5	12.4	28.4	11449	data imputed	2426	47.0	90,082
Sierra Leone	63	2.42	22.6	25.5	7.4	17.5	359	50.4	2428	62.6	5,989,641
Somalia	data imputed	6.05	22.9	26.1	7.0	17.6	225	data imputed	1762	63.9	11,080,122
South Africa	279	5.52	27.1	51.2	26.1	24.3	6002	79.4	2986	39.8	49,119,766
Sudan	222	15.67	24.1	24.8	6.4	18.7	1445	69.7	2266	57.4	32,360,619
Swaziland	261	3.94	26.5	36.2	14.9	18.6	3047	49.3	2307	75.4	1,042,651
Tanzania	89	5.75	22.9	25.1	6.9	17.7	527	45.2	2017	75.0	40,681,416
Togo	76	6.15	23.2	25.8	7.1	18.4	443	45.5	2146	58.7	5,920,360
Tunisia	330	8.52	26.0	59.1	24.6	28.3	3778	85.2	3312	33.9	10,304,729
Uganda	84	2.50	22.1	20.4	4.3	14.9	492	43.2	2247	87.2	29,486,335
Zambia	90	3.94	22.3	25.6	6.8	16.8	1105	33.7	1885	64.8	12,502,958
Zimbabwe	216	1.82	23.7	36.6	14.3	20.1	528	55.3	2207	63.2	12,255,920

Supplement: Table 2: Characteristic variables by country for Asia, including 48 countries

Country	Per capita sugar intake [kcal/d]	Diabetes prevalence [%]	Mean BMI [kg/m²]	Overweight prevalence [%]	Obesity prevalence [%]	Mean age [years]	Per capita GDP [US$]	Per capita fat intake [g/d]	Total energy [kcal/d]	Rural population [%]	Total population
Afghanistan	73	9.59	22.9	20.5	4.4	17.6	390	32.1	data imputed	76.4	27,100,542
Armenia	314	7.11	26.1	52.3	18.3	30.8	3347	76.6	2250	36.2	2,932,615
Azerbaijan	140	7.11	26.6	50.9	17.7	27.6	3788	48.0	2996	48.3	8,724,237
Bahrain	data imputed	16.52	24.9	63.5	27.6	29.7	20977	data imputed	data imputed	11.6	1,035,924
Bangladesh	55	8.38	21.3	17.6	2.8	22.5	543	28.6	2250	73.4	142,660,381
Bhutan	data imputed	9.75	23.5	24.2	5.2	20.5	1741	data imputed	data imputed	66.7	664,873
Brunei	305	12.79	26.7	37.9	12.1	27.2	36202	79.6	2987	25.7	374,967
Cambodia	166	4.00	21.8	19.4	3.1	21.3	632	35.9	2245	79.1	13,679,953
China	59	9.74	23.4	29.3	5.0	33.2	2657	86.6	2974	57.4	1,345,993,891
Cyprus	273	9.24	26.8	57.7	20.4	35.1	31495	112.3	3199	30.3	1,063,708
Dem. People's Rep. Korea	48	4.00	23.8	30.5	5.9	32.4	597	34.0	2146	37.7	24,188,330
Georgia	418	7.11	27.1	51.4	19.3	38.0	2317	67.0	2813	47.4	4,166,860
Hong Kong	282	8.33	data imputed	data imputed	data imputed	41.2	30673	133.9	data imputed	data imputed	6,840,017
India	175	10.39	21.5	17.5	3.1	24.8	1004	50.8	2301	70.8	1,183,209,471
Indonesia	127	6.32	22.7	24.9	5.5	26.9	1975	52.5	2535	49.6	232,374,239
Iran	262	9.59	25.9	58.8	23.3	25.8	4857	73.1	3042	32.0	71,336,476
Iraq	189	8.83	28.3	62.3	28.0	20.0	2639	53.9	data imputed	33.4	27,911,242
Israel	224	6.74	27.0	63.0	24.8	29.9	25820	146.1	3540	8.4	6,847,148
Japan	194	5.72	22.7	25.7	3.6	43.5	35137	89.0	2806	33.7	128,494,050
Jordan	470	11.75	28.7	67.4	33.1	23.5	2792	95.2	2977	21.6	6,255,290
Kazakhstan	313	7.11	26.1	51.2	19.0	29.1	6619	117.0	3359	42.5	15,702,112
Korea	176	6.80	23.6	28.9	4.1	35.8	22883	90.4	3074	18.8	49,034,813
Kuwait	352	15.84	29.2	71.7	35.6	26.0	45792	123.5	3038	1.7	2,504,026
Kyrgyzstan	233	7.11	26.1	45.5	14.4	23.9	733	61.5	2672	63.9	5,184,384

Lao	46	4.00	22.4	22.1	4.1	19.0	768	33.4	2227	70.3	5,944,950
Lebanon	372	12.71	27.3	65.9	29.7	28.3	6087	111.1	3107	13.1	4,767,347
Malaysia	373	16.74	25.3	38.7	13.1	24.4	7269	83.8	2908	30.4	26,720,367
Maldives	336	data imputed	24.9	26.7	6.7	18.1	5560	60.3	data imputed	data imputed	335,172
Mongolia	116	4.82	25.7	52.3	17.9	24.6	1895	84.2	2254	43.0	2,593,819
Myanmar	5	4.61	22.3	21.9	4.6	27.4	371	57.5	2438	68.1	49,621,479
Nepal	46	7.26	22.0	18.7	3.3	20.5	418	43.1	2349	83.3	26,382,586
Oman	311	12.61	26.1	59.6	24.3	18.9	15805	75.7	data imputed	28.5	2,657,162
Pakistan	257	8.35	23.6	25.6	7.1	20.9	949	73.4	2251	64.3	167,808,106
Philippines	226	7.07	22.9	25.3	5.4	22.7	1673	50.5	2518	35.8	89,405,482
Qatar	data imputed	16.52	28.7	69.5	32.4	31.9	67006	data imputed	data imputed	4.4	1,218,441
Saudi Arabia	309	17.72	28.2	67.5	32.8	21.4	16472	data imputed	3133	18.6	25,184,589
Singapore	data imputed	10.99	23.6	30.7	5.6	37.8	38031	data imputed	data imputed	data imputed	4,578,629
Sri Lanka	262	10.68	22.7	20.7	4.1	30.0	1869	45.8	2392	84.9	19,842,044
Syria	data imputed	8.23	27.9	58.5	25.1	21.1	2051	data imputed	3049	46.2	19,878,257
Tajikistan	156	7.11	25.5	42.5	12.2	21.3	520	57.5	2127	73.6	7,062,667
Thailand	353	7.04	24.0	28.6	7.9	32.4	3972	62.4	2529	67.1	66,182,064
Timor-Leste	73	6.86	20.9	19.0	2.9	21.1	2705	39.5	2016	73.1	1,036,388
Turkey	266	12.13	27.6	64.4	29.5	28.6	9709	113.5	3482	31.8	69,581,854
Turkmenistan	80	7.11	26.1	48.9	16.3	22.3	3796	79.1	2754	51.8	4,870,142
United Arab Emirates	311	17.26	28.1	65.4	29.0	30.1	43230	83.8	3138	22.2	6,168,846
Uzbekistan	199	7.57	26.3	45.5	14.4	22.9	819	67.2	2525	63.3	27,204,711
Vietnam	107	6.00	21.3	15.9	1.6	26.4	901	61.3	2769	72.7	85,419,588
Yemen	250	5.35	23.4	45.4	14.6	16.7	1178	47.8	2032	69.9	21,282,514

Supplement: Table 3: Characteristic variables by country for Europe, including 40 countries

Country	Per capita sugar intake [kcal/d]	Diabetes prevalence [%]	Mean BMI [kg/m²]	Overweight prevalence [%]	Obesity prevalence [%]	Mean Age [years]	Per capita GDP [US$]	Per capita fat intake [g/d]	Total energy [kcal/d]	Rural population [%]	Total population
Albania	189	10.08	26.3	54.8	19.3	29.2	3531	91.3	2904	53.9	3,033,993
Andorra	data imputed	7.97	26.8	62.9	24.8	41.5	48580	data imputed	data imputed	data imputed	82,682
Austria	412	6.35	25.5	52.6	18.4	41.3	46762	162.8	3760	33.1	8,313,731
Belarus	288	5.18	26.4	57.7	23.0	38.2	4908	118.4	3086	27.0	9,480,514
Belgium	447	4.29	26.0	58.3	20.7	41.1	44102	165.2	3690	2.7	10,697,576
Bosnia and Herzegovina	136	10.08	25.5	51.2	16.3	38.9	4181	70.0	3084	53.1	3,762,791
Bulgaria	308	5.81	26.1	59.8	23.2	40.9	5869	93.8	2761	29.2	7,576,675
Croatia	330	5.59	26.9	57.5	22.5	40.6	13780	119.9	2987	43.1	4,362,427
Czech Republic	288	6.82	26.9	60.9	24.5	39.5	18271	134.3	3317	26.5	10,357,535
Denmark	479	6.41	25.3	54.0	18.1	40.1	58396	130.4	3397	13.6	5,469,920
Estonia	427	4.02	26.2	54.4	20.1	39.4	16541	93.5	3129	30.6	1,344,295
Finland	297	5.76	26.0	56.6	20.7	41.6	48201	131.7	3215	37.0	5,297,741
France	327	4.77	25.0	57.7	20.1	39.0	41504	160.4	3553	22.9	61,869,229
Germany	340	8.31	26.4	55.3	20.7	43.0	42287	146.0	3530	26.5	81,277,836
Greece	270	4.55	27.1	60.6	23.2	41.2	27984	149.3	3700	39.3	11,120,371
Hungary	158	7.55	27.0	59.5	24.5	38.9	13952	139.6	3438	32.9	10,024,158
Iceland	365	5.31	26.1	57.8	20.3	34.5	70495	151.3	3330	7.8	305,181
Ireland	275	3.28	27.2	58.3	22.8	34.3	61369	129.5	3532	39.0	4,324,649
Italy	270	4.78	25.7	56.8	18.7	42.5	37141	156.4	3657	32.1	58,747,862
Latvia	200	4.91	26.4	56.5	22.4	39.6	14056	124.2	3019	32.0	2,198,089
Lithuania	295	3.67	26.4	58.2	25.0	38.6	12187	103.2	3419	33.2	3,258,984
Luxembourg	112	4.42	26.2	57.1	20.9	38.9	107190	145.1	3685	17.5	474,917
Macedonia	337	10.08	26.4	56.3	20.8	34.4	4036	120.6	2983	33.6	2,065,408
Malta	461	8.83	27.1	65.1	27.5	39.0	19327	109.3	3592	5.9	407,854
Moldova	289	5.72	26.8	50.0	17.5	34.0	1066	75.8	2907	58.1	4,130,129

Montenegro	229	10.08	26.2	57.5	21.6	data imputed	5941	118.0	2445	39.6	619,554
Netherlands	434	5.29	25.5	56.0	18.6	39.7	51338	131.6	3243	18.7	16,506,652
Norway	336	5.31	26.5	56.6	21.3	38.7	84982	148.3	3455	22.6	4,719,403
Poland	417	5.91	26.3	56.5	21.5	37.3	11194	111.9	3397	38.7	38,353,339
Portugal	239	9.85	25.8	55.4	19.0	38.8	22592	139.5	3583	41.1	10,573,150
Romania	250	9.74	26.3	55.6	20.7	36.9	8364	106.8	3510	46.0	21,034,196
Russia	418	6.18	26.1	55.6	21.9	38.2	9168	94.0	3272	27.2	143,266,207
Serbia	282	10.08	25.9	55.2	20.0	37.3	5458	82.2	2729	48.2	9,101,189
Slovakia	322	7.29	26.2	54.5	19.1	36.1	14249	101.1	2885	43.6	5,398,693
Slovenia	170	7.25	26.2	54.4	18.8	41.0	23894	119.6	3220	51.2	2,012,128
Spain	281	7.17	26.1	60.2	22.4	40.3	32587	147.3	3271	23.0	45,429,071
Sweden	382	4.79	25.9	54.8	19.0	41.1	53300	121.9	3116	15.5	9,162,941
Switzerland	535	5.59	25.1	52.8	18.0	40.4	63479	155.7	3421	26.6	7,536,986
Ukraine	445	7.11	26.4	56.8	22.7	39.2	3206	94.9	3230	32.1	46,366,347
United Kingdom	331	4.28	27.0	61.7	25.4	39.6	50221	140.8	3442	10.3	61,455,154

Supplement: Table 4: Characteristic variables by country for North and Central America, including 23 countries

Country	Per capita sugar intake [kcal/d]	Diabetes prevalence [%]	Mean BMI [kg/m²]	Overweight prevalence [%]	Obesity prevalence [%]	Mean Age [years]	Per capita GDP [US$]	Per capita fat intake [g/d]	Total energy [kcal/d]	Rural population [%]	Total population
Antigua and Barbuda	278	13.17	26.3	45.7	17.1	30.3	14351	84.9	2319	69.8	84,029
Bahamas	314	13.17	27.7	62.3	29.5	28.1	31018	92.9	2701	16.5	337,387
Barbados	515	13.57	27.5	49.8	20.9	35.0	16392	90.6	3055	60.4	278,701
Belize	417	17.11	28.6	52.4	22.0	19.9	4325	68.4	2719	48.8	299,031
Canada	398	7.37	26.9	62.2	27.1	39.1	44366	144.9	3530	19.7	32,930,795
Costa Rica	519	8.78	27.0	58.4	22.9	26.8	6121	87.5	2813	37.2	4,404,626
Cuba	488	8.27	25.4	56.2	22.6	36.3	5184	62.2	3295	24.4	11,251,117
Dominica	383	11.62	26.7	57.7	25.6	29.1	6042	79.5	3115	26.9	70,797
Dominican Republic	359	8.20	26.1	57.6	24.5	24.5	4606	83.1	2263	31.7	9,338,856
El Salvador	380	8.87	27.3	57.4	22.2	22.0	2796	63.2	2585	39.7	6,105,810
Grenada	351	10.71	26.6	48.7	19.1	22.1	7324	87.9	data imputed	data imputed	105,183
Guatemala	532	10.18	26.2	53.2	18.9	18.9	2490	58.8	2171	51.9	13,700,326
Haiti	205	6.65	24.8	50.7	19.4	18.4	625	37.3	1848	54.4	9,495,336
Honduras	367	7.21	26.3	52.8	19.0	19.7	1604	73.5	2601	52.6	7,808,520
Jamaica	405	11.28	27.2	52.6	22.3	23.2	4612	87.3	2848	47.0	2,768,229
Mexico	425	13.06	27.7	62.8	26.8	25.6	9413	91.1	3245	23.1	109,170,503
Nicaragua	356	11.47	26.9	55.7	21.5	21.3	1344	60.5	2400	43.6	5,590,066
Panama	269	8.33	26.6	56.4	20.6	26.4	6166	63.5	2451	27.5	3,453,671
St. Kitts and Nevis	378	12.84	28.9	49.4	20.4	28.1	13543	80.2	2452	data imputed	47,769
St. Lucia	389	11.62	29.1	45.3	17.4	25.6	7635	82.1	2744	data imputed	167,644
St. Vincent and the Grenadines	434	11.62	26.8	52.0	21.2	27.4	6276	76.8	2806	data imputed	108,516
Trinidad and Tobago	586	10.97	27.2	43.1	16.3	31.8	16484	84.1	2713	87.1	1,308,450
USA	302	10.79	28.6	65.8	33.6	36.6	48077	163.2	3770	18.6	300,608,425

Supplement: Table 5: Characteristic variables divided by country for Oceania, including 16 countries

Country	Per capita sugar intake [kcal/d]	Diabetes prevalence [%]	Mean BMI [kg/m²]	Overweight prevalence [%]	Obesity prevalence [%]	Mean age [years]	Per capita GDP [US$]	Per capita fat intake [g/d]	Total energy [kcal/d]	Rural population [%]	Total population
Australia	396	5.07	27.0	62.7	26.7	37.1	46968	143.9	3186	11.4	20,916,339
Cook Islands	data imputed	data imputed	32.6	83.4	53.8	25.3	11682	data imputed	data imputed	data imputed	18,994
Fiji	325	14.49	27.3	61.3	27.7	24.9	4047	95.1	3033	48.2	836,185
Kiribati	456	22.66	29.7	77.0	43.5	20.4	1378	100.8	data imputed	data imputed	96,531
Marshall Islands	data imputed	data imputed	29.4	82.1	50.7	20.7	2893	data imputed	data imputed	data imputed	56,046
Micronesia	data imputed	data imputed	29.4	73.8	42.9	21.2	2442	100.8	data imputed	data imputed	104,442
Nauru	data imputed	24.07	32.7	87.8	59.6	21.0	2311	data imputed	data imputed	data imputed	9,846
New Caledonia	223	23.36	data imputed	data imputed	data imputed	28.1	36681	115.1	2789	35.4	243,332
New Zealand	531	8.08	27.8	63.7	28.4	34.2	32439	121.0	3150	13.6	4,233,046
Palau	data imputed	15.89	29.5	83.7	53.1	32.0	9600	data imputed	data imputed	data imputed	19,159
Papua New Guinea	data imputed	17.65	25.2	50.1	19.0	21.4	1440	data imputed	data imputed	87.5	6,808,503
Samoa	244	9.21	31.7	75.9	44.7	20.4	3530	129.3	2878	77.1	182,045
Solomon Islands	72	18.68	25.9	51.9	19.9	19.1	1193	47.5	2434	82.5	492,133
Tonga	data imputed	15.42	31.9	76.7	45.4	21.3	2980	data imputed	data imputed	75.7	102,577
Tuvalu	data imputed	27.25	30.5	79.9	48.6	24.9	2637	data imputed	data imputed	data imputed	10,219
Vanuatu	177	12.02	26.0	54.3	22.6	23.4	2393	100.8	2722	75.9	219,464

Supplement: Table 6: Characteristic variables by country for South America, including 12 countries

Country	Per capita sugar intake [kcal/d]	Diabetes prevalence [%]	BMI mean [kg/m²]	Overweight prevalence [%]	Obesity prevalence [%]	Age [years]	Per capita GDP [US$]	Per capita fat intake [g/d]	Total energy [kcal/d]	Rural population [%]	Total Population
Argentina	404	5.50	27.3	60.7	26.3	29.9	7249	110.8	3001	8.2	39,684,303
Bolivia	257	6.89	26.1	53.7	18.3	22.2	1390	49.8	2093	34.8	9,558,438
Brazil	385	8.11	26.1	54.0	20.1	28.6	7314	107.0	3099	14.8	190,130,445
Chile	442	8.46	27.6	61.0	26.1	30.7	10527	88.2	2957	11.8	16,530,201
Colombia	336	7.44	25.9	56.8	20.4	26.6	4674	75.9	2662	25.8	43,737,512
Ecuador	162	5.55	26.7	53.7	18.1	23.9	3591	89.1	2304	35.0	14,296,554
Guyana	338	11.62	26.0	46.6	17.9	27.8	2327	56.8	2753	71.7	746,477
Paraguay	239	8.27	26.3	50.8	18.2	21.6	2312	89.4	2622	40.3	5,998,430
Peru	196	5.95	26.2	55.3	18.1	25.5	3612	41.9	2426	28.7	28,333,050
Suriname	436	12.54	26.3	56.7	24.4	27.1	5761	74.9	2468	25.5	511,181
Uruguay	313	6.93	26.9	60.8	26.0	32.9	7010	95.5	2818	7.8	3,331,753
Venezuela	386	6.47	26.6	61.8	24.0	24.9	8319	76.9	2582	6.9	27,247,601

Supplement: Table 7: Characteristic variables by region, including 192 countries

Region	Per capita sugar intake [kcal/d]	Diabetes prevalence [%]	Mean BMI [kg/m²]	Overweight prevalence [%]	Obesity prevalence [%]	Mean age [years]	Per capita GDP [US$]	Per capita fat intake [g/d]	Total energy [kcal/d]	Rural population [%]	Total population
World	194	8.46	24.0	35.2	11.2	28.5	8169	80.4	2740	51.3	6,662,675,376
Africa	148	6.10	23.6	32.1	11.1	19.4	1590	54.1	2445	61.0	953,240,473
Eastern Africa	96	5.03	22.0	22.7	5.6	17.7	494	35.5	2031	77.6	302,739,252
Middle Africa	109	5.95	22.4	24.8	6.7	17.0	1272	49.6	2129	58.0	119,442,036
Northern Africa	283	12.82	26.8	53.9	23.4	23.7	2558	65.8	3020	49.0	192,202,904
Southern Africa	270	5.35	26.8	49.6	24.8	23.8	5693	75.5	2907	42.6	56,031,317
Western Africa	89	2.67	23.0	26.1	7.4	18.4	1341	59.9	2634	57.2	282,824,964
Asia	137	9.19	22.9	26.6	6.0	28.3	3405	67.3	2600	59.8	4,044,026,753
Central Asia	217	7.32	26.1	46.9	15.5	24.4	2535	79.5	2728	58.2	60,024,016
Eastern Asia	72	9.29	23.3	29.0	4.9	34.1	5579	86.2	2947	54.7	1,557,144,920
Southern Asia	174	9.91	21.9	20.2	4.4	24.1	1118	51.6	2287	69.1	1,639,339,651
South-Eastern Asia	158	6.30	22.6	23.6	5.0	26.6	1888	54.7	2550	55.6	575,338,106
Western Asia	262	10.98	27.3	61.0	26.7	24.8	9926	88.4	2647	34.7	212,180,060
Europe	350	6.05	26.1	57.2	21.5	39.2	25987	124.9	3366	27.9	731,519,557
Eastern Europe	386	6.58	26.3	56.3	22.0	38.2	8679	100.9	3275	31.7	295,987,793
Northern Europe	339	4.55	26.7	59.6	23.6	39.6	51738	136.6	3385	15.3	97,536,357
Southern Europe	269	6.55	26.0	57.7	20.4	40.6	29214	141.7	3414	33.0	151,318,480
Western Europe	374	5.22	25.3	56.6	19.7	39.8	45354	155.4	3526	21.6	186,676,927
Oceania	407	8.54	26.8	60.3	25.6	32.8	33987	136.7	2506	28.4	34,348,861
South America	353	7.44	26.3	55.6	20.9	27.9	6435	94.5	2899	18.2	380,105,945
North America	348	10.78	28.0	63.4	30.0	32.7	33497	133.2	3466	22.9	519,433,787
Carribean	371	8.25	25.7	54.3	22.0	26.9	4471	64.4	2555	38.7	35,362,014
Central America	428	12.16	27.4	60.8	25.1	24.5	7812	85.0	3040	28.9	150,532,553
Northern America	311	10.45	28.4	65.4	33.0	36.8	47711	161.4	3746	18.7	333,539,220

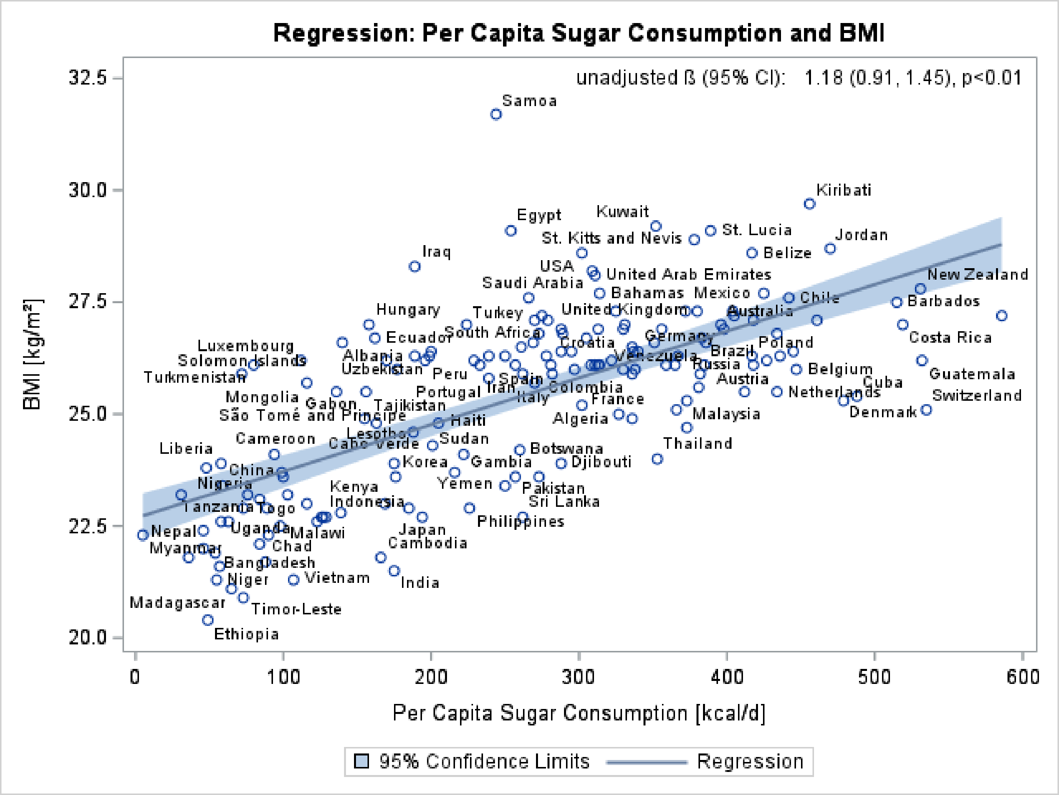

Supplement: Figure 2: Linear regression of per capita sugar consumption (2007 - FAO) and BMI (2012 – WHO) – including 192 countries

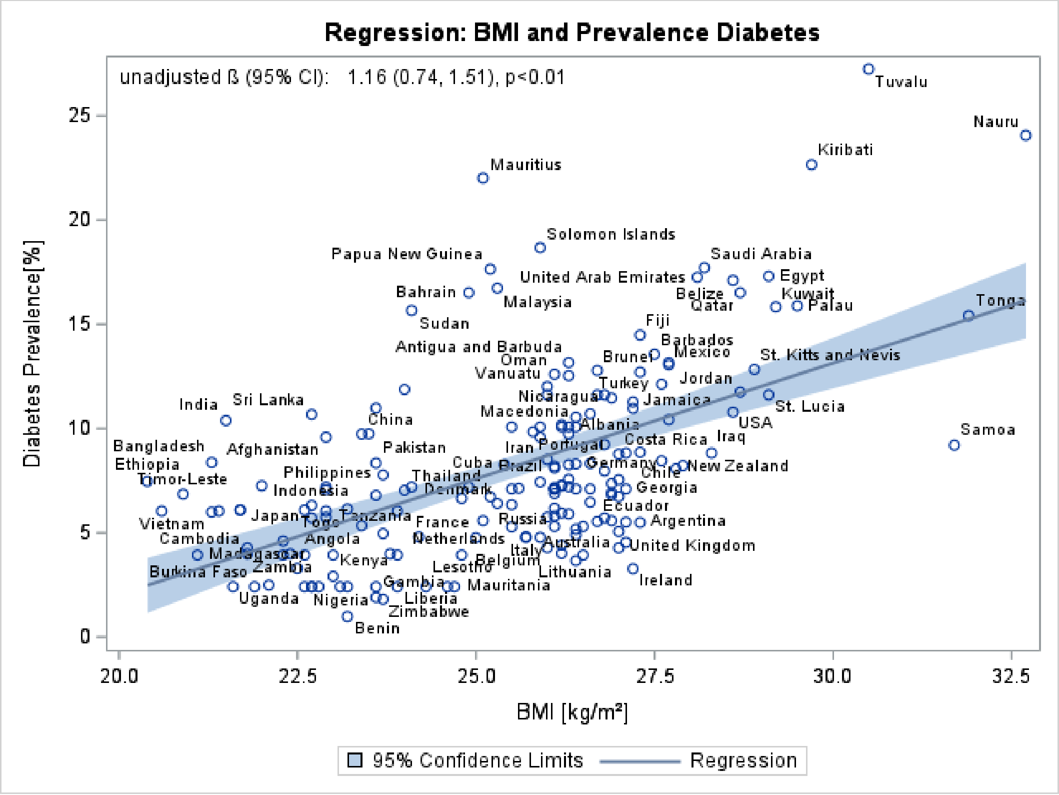

Supplement: Figure 3: Linear regression of BMI (2012 - WHO) and diabetes mellitus (2017 - IDF) – including 192 countries

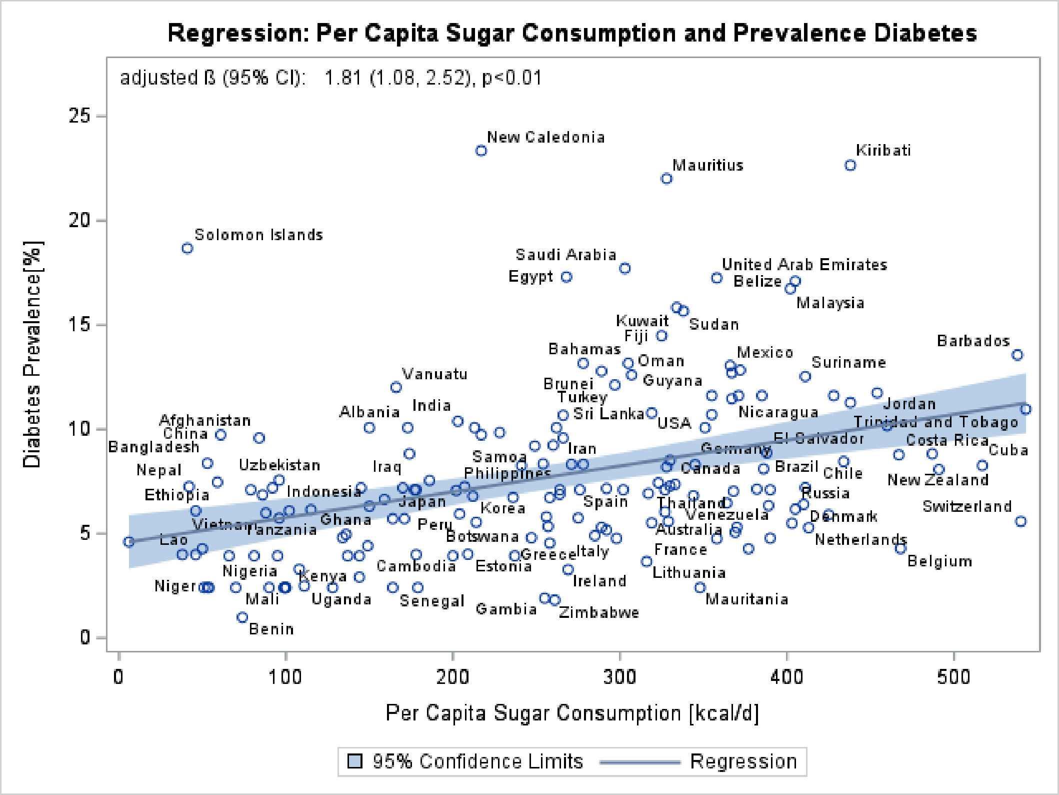

Supplement: Figure 4: Sensitivity analysis: Linear regression of per capita sugar consumption and diabetes mellitus using the most recent data for exposure (2013 - FAO), mediator (2016 - WHO) and outcome (2017 - IDF) – including 192 countries

Supplement: Table 8: Mediation associations of BMI per 100 kcal/day per capita sugar consumption on the association between per capita sugar consumption and diabetes prevalence: sensitivity analysis with most recent data for exposure (2013 - FAO), mediator (2016 - WHO) and outcome (2017 - IDF) – based on 192 countries

	Model 1 (crude)<br>β [95% CI]	Model 2 (adjusted)*<br>β [95% CI]
β_{total}	1.48 [0.89, 2.06]	1.81 [1.08, 2.52]
β_{direct}	0.18 [-0.65, 0.98]	0.65 [-0.19, 1.46]
$\beta_{indirect}$	1.29 [0.73, 2.00]	1.14 [0.64, 1.77]
% mediated	87.9% [46.1%, 100.0%**]	64.2% [33.5%, 100.0%**]

* adjusted for age [years] (2013 - CIA), per capita gross domestic product [US$] (2013 - UN), total energy [kcal/day] (2013 - FAO), per capita fat intake [g/d] (2013 - FAO) and rural population [%] (2013 - FAO)

** bootstrap 95% CI cut off by 100%

Supplement: Table 9: Mediation associations of overweight and obesity (2012 - WHO) per 100 kcal/day per capita sugar consumption on the association between per capita sugar consumption (2007 - FAO) and diabetes prevalence (2017 - IDF) – based on 192 countries

Mediator		Model 1 (crude) β [95% CI]	Model 2 (adjusted)* β [95% CI]
Overweight	β total	1.44 [0.94, 2.00]	1.68 [1.08, 2.36]
	β direct	0.64 [-0.22, 1.49]	0.99 [0.30, 1.74]
	β indirect	0.79 [0.19, 1.49]	0.67 [0.26, 1.16]
	% mediated	54.9% [12.9%, 100%**]	40.4% [15.5%, 77.6%]
Obesity	β total	1.44 [0.94, 2.00]	1.68 [1.08, 2.36]
	β direct	0.37 [-0.36, 1.06]	0.83 [0.17, 1.54]
	β indirect	1.07 [0.49, 1.74]	0.84 [0.38, 1.35]
	% mediated	74.6% [34.7%, 100.0%**]	50.6% [22.9%, 87.0%]

* adjusted for age [years] (2007 - CIA), per capita gross domestic product [US$] (2007 - UN), total energy [kcal/day] (2007 - FAO), per capita fat intake [g/d] (2007 - FAO) and rural population [%] (2007 - FAO)

** bootstrap 95% CI cut off by 100%

Supplement: Table 10: Correlation coefficients between per capita sugar consumption (2007 - FAO) and diabetes prevalence (2017 - IDF) divided by region – based on 192 countries

Continents	n	r (95% CI)	p Value
Africa	53	0.42 (-0.03, 0.73)	0.06
Asia	48	0.56 (0.17, 0.80)	0.01
Europe	40	-0.26 (-0.64, 0.23)	0.30
Oceania	16	-0.34 (-0.97, 0.86)	0.67
South America	12	0.46 (-0.60, 0.94)	0.41
North America	23	0.15 (-0.50, 0.70)	0.67

Supplement: Table 11: Correlation coefficients between per capita sugar consumption (2007 - FAO) and diabetes prevalence (2017 - IDF) divided income classification according to the UN – based on 192 countries

Countries based on income	n	r (95% CI)	p Value
Low income countries	36	-0.16 (-0.62, 0.38)	0.57
Lower middle income countries	53	0.41 (-0.05, 0.73)	0.08
Upper middle income countries	53	0.35 (-0.04, 0.65)	0.08
High income countries	50	-0.03 (-0.44, 0.40)	0.91

Supplement: Table 12: Correlation coefficients between per capita sugar consumption (2007 - FAO), diabetes prevalence (2017 - IDF), mean BMI (2012 - WHO), mean age (2007 - CIA), per capita GDP (2007 - UN), per capita fat intake (2007 - FAO), total energy (2007 - FAO), rural population (2007 - FAO) and overweight and obesity prevalence (2012 - WHO) – based on 192 countries

Variables		r (95% CI)	p Value
Per capita sugar consumption	Diabetes prevalence	0.37 (0.16, 0.54)	<0.01
Per capita sugar consumption	Mean BMI	0.67 (0.52, 0.78)	<0.01
Per capita sugar consumption	Mean age	0.48 (0.29, 0.63)	<0.01
Per capita sugar consumption	Per capita GDP	0.33 (0.11, 0.52)	<0.01
Per capita sugar consumption	Per capita fat intake	0.52 (0.34, 0.66)	<0.01
Per capita sugar consumption	Total energy	0.53 (0.34, 0.67)	<0.01
Per capita sugar consumption	Rural population	-0.57 (-0.72, -0.37)	<0.01
Per capita sugar consumption	Overweight prevalence	0.67 (0.53, 0.78)	<0.01
Per capita sugar consumption	Obesity prevalence	0.65 (0.50, 0.76)	<0.01
Mean BMI	Diabetes prevalence	0.58 (0.41, 0.71)	<0.01
Mean age	Diabetes prevalence	0.02 (-0.18, 0.22)	0.82
Per capita GDP	Diabetes prevalence	0.01 (-0.20, 0.22)	0.91
Per capita fat intake	Diabetes prevalence	0.12 (-0.09, 0.33)	0.26
Total energy	Diabetes prevalence	0.17 (-0.04, 0.36)	0.11
Rural population	Diabetes prevalence	-0.13 (-0.34, 0.10)	0.27
Overweight prevalence	Diabetes prevalence	0.48 (0.29, 0.64)	<0.01
Obesity prevalence	Diabetes prevalence	0.57 (0.38, 0.71)	<0.01
